# Biogenesis of ZnO nanoparticles using *Pandanus odorifer* leaf extract: anticancer and antimicrobial activities

**DOI:** 10.1039/c9ra01659g

**Published:** 2019-05-16

**Authors:** Afzal Hussain, Mohammad Oves, Mohamed F. Alajmi, Iqbal Hussain, Samira Amir, Jahangeer Ahmed, Md Tabish Rehman, Hesham R. El-Seedi, Imran Ali

**Affiliations:** Department of Pharmacognosy, College of Pharmacy, King Saud University Riyadh 11451 Kingdom of Saudi Arabia malajmii@ksu.edu.sa; Center of Excellence in Environmental Studies, King Abdulaziz University Jeddah 21589 Kingdom of Saudi Arabia; Department of General Studies, Jubail Industrial College Jubail Industrial City Jubail 31961 Kingdom of Saudi Arabia; Department of Chemistry, College of Science & General Studies, Alfaisal University Riyadh 11451 Kingdom of Saudi Arabia; Department of Chemistry, College of Science, King Saud University Riyadh 11451 Kingdom of Saudi Arabia; Pharmacognosy Group, Department of Medicinal Chemistry, Uppsala University, Biomedical Centre Box 574 751 23 Uppsala Sweden; Department of Chemistry, College of Sciences, Taibah University Al-Medina Al-Munawara 41477 Saudi Arabia drimran.chiral@gmail.com; Department of Chemistry, Jamia Millia Islamia Central University New Delhi India

## Abstract

The continuously increasing incidence rates of cancer and infectious diseases are open threats to the sustainable survival of animals and humans. In the last two decades, the demands of nanomaterials as modern therapeutic agents have increased. In this study, biogenic zinc oxide nanoparticles (ZnO NPs) were developed from aqueous *Pandanus odorifer* leaf extract (POLE) and characterized using modern methods and tools, such as electron microscopy, X-ray diffraction, energy dispersive X-ray spectroscopy (EDX), Fourier transform infrared spectroscopy and UV-vis spectroscopy, which indicated the formation of very pure, spherical NPs approximately 90 nm in size. The anticancer activity of the ZnO NPs was evaluated by MTT and neutral red uptake (NRU) assays in MCF-7, HepG2 and A-549 cells at different doses (1, 2, 5, 10, 25, 50, 100 μg ml^−1^). Moreover, the morphology of the treated cancer cells was examined by phase contrast microscopy. The results suggest that the synthesized ZnO NPs inhibited the growth of the cells when applied a concentration from 50–100 μg ml^−1^. Moreover, the biogenic ZnO NPs were analysed as an antimicrobial agent against pathogenic bacteria. The highest antibacterial activity was observed against Gram-positive *Bacillus subtilis* (26 nm) and Gram-negative *Escherichia coli* (24 mm) at 50 μg per well. Complete bacterial growth (100%) vanished 100% upon treatment with ZnO NPs at 85 μg ml^−1^. Overall, POLE mediated derived biogenic ZnO NPs could serve as a significant anticancer and antimicrobial agent and be used in the development of novel drugs and skin care products.

## Abstract 2

The continuously increasing incidence rates of cancer and infectious diseases are open threats to the sustainable survival of animals and humans.

## Graphical abstract

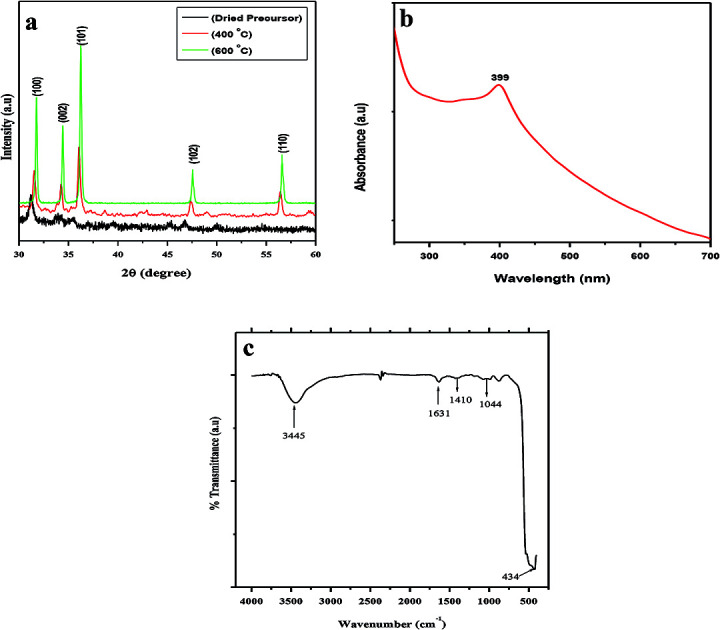

## 1. Introduction

Nanomaterials are extensively used in diverse fields, such as energy, food processing, agriculture, and innovative textile fabrication, as well as several biomedical applications (biosensors, nanomedicine, and bionanotechnology).^1,2^ It is known that nanoscale materials can be suitable agents for influencing the properties and functions of living and anthropogenic systems.^3^ Nanotechnology plays a vital role in nanomedicine because nano structures of different shapes exhibit new and considerably enhanced physicochemical and biological properties as well as distinct phenomena and functionalities.^4^ The intrinsic properties of metal and metal oxide nanoparticles (NPs) such as zinc oxide (ZnO), titanium dioxide (TiO_2_), and silver are mostly characterized by the NP size, composition, crystallinity, and morphology. Reducing the size of a material to the nanoscale can modify its chemical, mechanical, electrical, structural, morphological, and optical properties. These changed features allow NPs to interact uniquely with cellular biomolecules and thus facilitate the physical transfer of NPs into intracellular structures.^5,6^ Among these NPs, ZnO NPs have gained tremendous interest due to their potential as chemotherapeutic and antimicrobial agents.^7,8^ Cancer is one of the main causes of human mortality worldwide, accounting for approximately 7.6 million deaths every year globally. It is estimated that the number of deaths may increase up to 11 million in 2030. Nearly, 70% of human deaths occur from cancer-related disease in the poor or middle-income countries because of the limited availability of preventive on, diagnostic, and therapeutic resources.^9–12^ In current reports of WHO cancer is responsible for apparently 9.6 million deaths in 2018 and about one in six deaths was happened due to cancer. Cancer is now the second leading cause of human death worldwide [https://www.who.int/news-room/fact-sheets/detail/cancer].

Surgical procedures are effective when the cancer is localized, although adjuvant chemotherapy is still needed. Radiotherapy plays a significant role in cancer treatment, provided that the cancer is not disseminated. Currently, many medications are being used alone or in combination with others, with the most useful medicines including cisplatin, carboplatin, bleomycin, 5-fluorouracil, doxorubicin, dactinomycin, 6-mercaptopurine, tamoxifen, taxol, topotecan, vinblastine, and etoposide,^13^ all of which are organically synthesized and extracted from plants. However, many anticancer agents are non-specific, resulting in elusive mechanisms of action, narrow spectra of activity, severe side effects (nausea, ear damage, vomiting, nephrotoxicity) and inherent or acquired resistance; these effects limit their successful clinical use.^14^ ZnO NPs have been effective in having minimal side effects and targeted action on cancer cells due to the large surface area of the NPs.^15–17^ At physiological pH, ZnO NPs are highly selective for cancer cells, resulting in the generation of active oxide, hydrogen peroxide (H_2_O_2_) and superoxide from their surface, which may be a source of cytotoxicity in cancer cells.^18–20^

Another important aspect with more acceptance is the development of nanomaterials with antimicrobial properties to overcome the phenomenon of multidrug resistance. Multidrug resistance in bacteria is a serious health issue associated with enormous social and economic burdens.^21^ The emergence of a new persistent bacterial strain is the direct consequence of the non-judicial use of antibiotics.^22^ The risk of infection with pathogenic strains is increasing due to the lack of proper medication systems and sterilization techniques and the improper handling and treatment of hazardous materials.^23^ For example, infections by *Shigella flexneri* cause 1.5 million deaths annually due to the contamination of food and drinks.^24^ Other bacterial species that contribute to antibiotic resistance include *Escherichia coli* O157:H, *Campylobacter jejuni*, *Staphylococcus aureus*, *Pseudomonas aeruginosa*, *Enterococcus faecalis*, Salmonella strains, and *Clostridium perfringens*. Recently, some nanomaterials have been employed as antimicrobial agents to prevent infection with pathogenic microbes. In this regard, ZnO NPs have the potential to exert their antimicrobial activity by rupturing the cell wall of microorganisms through the generation of Zn^2+^ and reactive oxygen species (ROS).^25–27^

To overcome both life-threatening issues, the development of NP-based drugs has become in great demand to cure cancer and fight bacteria. Conventional methods for the synthesis of NPs include microwave decomposition,^28^ simple wet chemistry routes,^29^ deposition processes, simple precipitation methods,^30^ hydrothermal synthesis,^31^ solvothermal methods,^32^ microwave hydrothermal methods,^33^ and hydrothermal techniques.^34^ However, these physiochemical methods are expensive, time and energy consuming and generate multiple hazardous chemicals by-products. Thus, there is a need for a “green chemistry” approach to NP synthesis that includes clean, non-toxic and environmentally friendly methods that can be applied in the ambient atmosphere. NPs synthesized *via* green synthetic routes are highly water-soluble, biocompatible and less toxic. Plant extracts are a very promising tool for the facile green synthesis of NPs. *Citrus aurantifolia* fruit juice, *Parthenium hysterophorus* leaf extracts, and Aloe species extracts have been used in the synthesis of ZnO NPs.^35–37^*Pandanus odorifer* (Forssk.) Kuntze (synonym *Pandanus odoratissimus* Linn., Family: Pandanaceae) is a traditional Indian Ayurvedic medicine widely used for the treatment of headache, rheumatism, cold/flu, epilepsy, leucoderma, ulcers, hepatitis, smallpox, leprosy, syphilis, and even cancer. It also acts as a cardiotonic, antioxidant, dysuric, an aphrodisiac. The phytochemical analysis shows that it is a rich source of phytochemicals, such as lignans and isoflavones, coumestrol, alkaloids, steroids, carbohydrates, phenolic compounds, glycosides, proteins, amino acids, and vitamins, in addition to other nutrients.^38^ We have reported the synthesis of ZnO NPs using *Pandanus odorifer* leaf water extract (POLE), as a bio-template that never been reported. Like many plants, *Pandanus odorifer* leaf extract contains high levels of flavonoids and phenols. The quantified data of flavonoids/phenolic components present in the leaf extract of *Pandanus odorifer* has been given in the manuscript under the section phytochemical analysis of the plant extract. Moreover, the presence of hydroxyl and ketonic groups has been confirmed by FTIR analysis. It is a well-established fact that these functionally active components act as reducing as well as a stabilizing agent during the biosynthesis of metal-based nanoparticles. Recent studies have discovered that plant metabolites such as sugars, terpenoids, phenolic, alkaloids, phenolic acids, and proteins play a significant role in the reduction of metal ions into nanoparticles and in providing stability to nanoparticles. Moreover, the reducing power of a plant extract cannot be solely determined by a single bioactive component. Rather, it is the synergistic effect of all the bioactive components present in the plant extract to reduce a metal into nanoparticle.^39–41^ Previously we had reported the contents of *Pandanus odorifer* leaf extract (*i.e.* phenolic and flavonoid) that could trigger the nucleation and size of the nanoparticles.^42^

The synthesized ZnO NPs were characterized using modern techniques, such as X-ray diffraction (XRD), scanning electron microscopy (SEM), energy dispersive X-ray spectroscopy (EDX), Fourier transform infrared (FTIR) spectroscopy and UV-vis spectroscopy. The physical and morphological examinations of the ZnO NPs show the spherical structure. The well-defined nanocrystals were tested as an anticancer agent against MCF-7 (breast cancer), HepG2 (liver cancer), and A549 (human lung alveolar epithelial) cells. Simultaneously, these newly synthesized ZnO NPs were also used as an antimicrobial agent against Gram-positive (*B. subtilis*) and Gram-negative (*E. coli*) bacteria.

## 2. Experimental

### 2.1 Materials and reagents

Zinc acetate dihydrate {Zn (CH_3_COO)_2_·2H_2_O} was procured from Sigma Aldrich (USA). Bacterial culture media were purchased from HiMedia (Pvt. Ltd. Mumbai, India). Antibiotic/antimycotic solution, Dulbecco's Modified Eagle Medium (DMEM) and fetal bovine serum were procured from Invitrogen, Life Technologies, USA. Glassware and plastic consumables were obtained from Nunc, Denmark.

### 2.2 High pressurized solvent extraction (HPSE) for preparation of leaf extract

*Pandanus odorifer* plant leaves were collected from mature plants grown in the botanical garden of Aligarh Muslim University, Aligarh, U.P., India. For the preparation of the POLE, a specific speed extractor (Buchi, E-914, Germany) was used. The extraction cells were prepared by inserting a cellulose filter and metal frit at the bottom of each 10 ml stainless steel cell to prevent entering particles to the solvent lines and collection vials. Briefly, 5 g of freshly collected *Pandanus odorifer* leaves was cleaned, washed three times with ultrapure water, and further eroded by 70% ethanol in water to remove microorganisms contaminating the leaf surface. These leaves were cut into small pieces, dried in an oven at 50 °C overnight, and crushed into a fine powder. This powder was placed in the cell of the speed extractor, which was programmed to run for two cycles. Each cycle was fixed at 42 min, and the temperature was set at 50 °C. Initially, 100 ml of water was filtered, and concentrated water extract was obtained.^43^ This POLE was collected in tubes and stored at 4 °C.

### 2.3 Phytochemical analysis of the plant extract

Phytochemical analysis was conducted to identify the total phenolic and flavonoid contents of the POLE. The total phenolic content was estimated using a standard gallic acid curve, as previously described.^44^ Briefly, leaf extract (0.125 ml) was mixed with 0.5 ml of deionized water followed by the addition of 0.125 ml of Folin–Ciocalteu reagent and incubation for 5 min at room temperature. Then, 1.25 ml of Na_2_CO_3_ (7%) solution was added to the above mixture and made up to 3 ml with deionized water, and followed by incubation for 1.5 h at room temperature. The maximum absorption at 760 nm was monitored. The total flavonoid content was analysed using a standard quercetin curve, as previously described.^45^ Briefly, 0.5 ml of AlCl_3_ (2% in methanol) was mixed well with 0.5 ml of POLE and incubated for 10 min at room temperature; then absorbance at 368 nm was recorded.

### 2.4 Biogenesis of ZnO NPs

To prepare a reaction solution, 50 ml of 20 mM zinc acetate solution was added dropwise to 20 ml of POLE under constant stirring at 80 °C for 3 h. The reaction mixture became dark brown, and a brown precipitate developed. For further precipitation, the reaction mixture was kept overnight to allow complete reaction. The precipitate was obtained by centrifugation at 15 000 rpm for 10 min at room temperature (25 °C). The precipitate (containing zinc compound) was washed several times with ultrapure Milli-Q water to remove the unwanted biological and chemical moieties and then oven-dried at 70 °C for 24 h. Finally, the samples were calcined at various temperatures (400 and 600 °C) for 3 h before characterization.

### 2.5 Biophysical characterization of ZnO NPs

The phase purity of the ZnO NPs was characterized by XRD using a Phillips-PW 1729 X-ray diffractometer (Holland) with Cu radiation (1.54430 Å). The XRD patterns were recorded with a step size of 0.02° and a scan speed of 2°min^−1^ ranging from 30° to 80° of 2θ. The surface morphology of the resulting ZnO NPs was characterized by field emission scanning electron microscopy (FESEM) using a MIRA II LMH system. The UV-vis absorption spectrum of the ZnO NPs was recorded in the range of 300–800 nm using a UV-visible spectrophotometer (Evolution 201, Thermo Fisher Scientific). Distilled water was used as a reference. The involvement of organic functional groups in the nanomaterial formation was analysed by FTIR spectrometry (PerkinElmer), and the spectra of the product were recorded in the range of 4000–400 cm^−1^.

### 2.6 Anticancer activity and cell morphology

#### 2.6.1 Cell culture

The MCF-7 (breast cancer), HepG2 (liver cancer), and A-549 (lung cancer) cells were used to determine the cell viability against ZnO NPs exposure. The MCF-7, HepG2, and A-549 cells were obtained from American Type Culture Collection (ATCC) (Manassas, VA) USA. MCF-7, HepG2, and A-549 cells were cultured in DMEM in the presence of foetal bovine serum (10%), sodium bicarbonate (0.2%), and antibiotic/antimycotic solution (1 ml/100 ml of medium). The cells were maintained in a 5% CO_2_ and 95% atmosphere under high humidity at 37 °C. Each cell culture was assessed for viability by trypan blue dye exclusion assay,^46^ and batches showing > 98% cell viability were used in this study. MCF-7, HepG2, and A-549 cells were treated with varying concentrations (1–100 μg ml^−1^) of biogenic ZnO NPs. Each treated cell culture was deliberated used for cytotoxicity assays (MTT and NRU) or the morphology analysis.

#### 2.6.2 MTT assay for cytotoxicity

MTT {3-(4,5-dimethylthiazol-2-yl)-2,5-diphenyl tetrazolium bromide} reagent was used to evaluate cell viability.^47^ Briefly, from each treated cell culture, approximately 1 × 10^4^ cells were allowed to incubate in a CO_2_ chamber for 24 h at 37 °C in 96-well culture plates. Various doses of ZnO NPs (1–100 μg ml^−1^) were used to treat the cancer cells. After nanomaterial exposure, 10 μl per well MTT (reagent 5 mg ml^−1^ of stock in PBS) was added to 100 μl of cell suspension, and the plate was incubated for 4 h. Then, the supernatant was discarded, and 200 μl of DMSO was added to each well and mixed gently. The absorbance at 550 nm of the plates were maintained on a rocking shaker for 10 min at room temperature; the developed color was recorded using a multi-well microplate reader (Multiskan Ex, Thermo Fisher Scientific, Finland). Identical conditions were used for the untreated cell that served as the controls.

#### 2.6.3 Neutral red uptake (NRU) assay for cytotoxicity

The assessment of cytotoxicity by NRU assay was performed as previously described.^48^ After the treatment with ZnO NPs, the medium was extracted, and the cells were washed three times with PBS. The treated and washed cells were cultured in DMEM supplemented with NR (50 μg ml^−1^) and incubated for 3 h. Then, the medium was washed off rapidly with a solution containing 1% calcium chloride and 0.5% formaldehyde, and the cells were exposed to a mixture of ethanol (50%) and acetic acid (1%) for 20 min at 37 °C for dye extraction. The at 550 nm was measured, and the experimental values obtained were compared with the control values of the culture plate.

#### 2.6.4 Morphological examination of cells by phase-contrast microscopy

After treatment, morphological changes in the cellular structure were analysed. In this study, alterations in the (MCF-7, HepG2, and A-549) cellular structure were induced by ZnO NPs. Each type of cell was exposed to different concentrations (1–100 μg ml^−1^) of ZnO NPs. After washing cells from each treatment group were observed using an inverted phase-contrast microscope at a magnification of 20×.

#### 2.6.5 Cancer cell cytotoxicity analysis by flow cytometry

The death of MCF-7, HepG2, and A549 cancer cells was examined by double staining with Annexin V-FITC and propidium iodide (PI) according to the manufacturer's instructions.^49^ Both the Annexin V-FITC and PI kits were procured from Molecular Probe (Eugene, USA). Briefly, 1 × 10^6^ cells per ml were exposed to ZnO NPs (100 μg ml^−1^) for 48 h in 6-well plates under optimum conditions in a CO_2_ incubator. At the end of the specific period of exposure, the cells were trypsinized, washed with cold PBS and centrifuged at 1000 rpm for 10 min at 4 °C. The cell pellet was washed with PBS and re-suspended in 100 μl of 1× binding buffer (1 × 10^6^ cells per ml). Then, 5 μl of Annexin V-FITC and PI reagent was added to each cell suspension, and the cells were gently vortexed. Subsequently, the cells were incubated for 20 min at 25 °C in the dark. Each sample was then diluted by the addition of 400 μl of 1× binding buffer and examined. The fluorescence emission from the Annexin-V and PI-stained cells was measured at 530–575 nm using a flow cytometer (MACS Quant, Germany). Finally, each treated sample was subjected to flow cytometry on a FACS Calibur system and the data were analysed using BD Cell Quest™ Pro software (version 5.2). According to the software, the cells are represented as follows: lower right quadrant, early apoptotic cells (FITC^+^/PI^−^); lower left quadrant, normal cells (FITC^−^/PI^−^); upper right quadrant, late apoptotic cells (FITC^+^/PI^+^); upper left quadrant, necrotic cells (FITC^−^/PI^+^).

### 2.7 Antibacterial activity

#### 2.7.1 Bacterial cell viability in the presence of ZnO NPs

The antibacterial activity of the green synthesized ZnO NPs against Gram-positive, and Gram-negative bacteria was determined using *Bacillus subtilis*: LN827668.1 and, *E. coli*: LN835288.1 respectively. Both the bacteria *B. subtilis* and *E. coli* were procured from the library of culture collection of the King Fahad Medical Research Center (KFMRC) at King Abdulaziz University, Jeddah, Saudi Arabia. The Gram-negative *E. coli* and the Gram-positive *B. subtilis* were grown in nutrient broth (HiMedia, Pvt., Ltd., Mumbai, India) under the optimum conditions with shaking in an incubator for overnight. Bacterial growth was measured by culture turbidity as a qualitative measurement and further confirmed by plate culture testing to determine the viability in the presence and absence of ZnO NPs. To determine the bacterial growth rate in the presence of the ZnO NPs, various (10, 20, 40, 60, 80 and 100 μg ml^−1^) doses were added to the liquid medium and incubated with the bacteria at 37 °C. Cultures of both bacteria without NPs were incubated in the same medium under the optimum growth conditions as a control. The same concentrations of NPs in separate media without bacteria were used as blank controls to account for optical interference by the light-scattering properties of the NPs. An overnight primary bacterial culture (1 ml) was used to inoculate 100 ml of nutrient broth (secondary culture) with different concentrations of ZnO NPs. The culture was incubated at 37 °C, and the optical density was monitored every hour with UV-vis spectrophotometer (Evolution 201, Thermo Fisher Scientific). Culture (0.1 ml) from each flask treated was spread on the media surface and incubated for 16 h. The number of colonies that appeared was counted as an indicator of the number of bacteria. These data were used to define the minimum inhibitory concentration and maximum bactericidal concentration of applied the ZnO NPs.

#### 2.7.2 Antibacterial activity of ZnO NPs determined by zone inhibition assay

In this study, the antibacterial properties of the biogenic ZnO NPs against *B. subtilis* and *E. coli* were examined. A fresh culture of both bacterial strains was grown in nutrient broth overnight at 35 °C on an orbital shaker at 120 rpm. Nutrient agar plates were also prepared separately. These plates were inoculated separately with fresh culture (100 μl) of *B. subtilis* and *E. coli* by the spread plate method. Furthermore, an 8 mm well was prepared on these inoculated plates using a sterilized steel borer, and each well base was sealed by molten agar to prevent surface leakage of the loaded material. Each well was filled with 100 μg of the biogenic ZnO NP suspension and incubated overnight, and zone formation around the loaded material was observed.

#### 2.7.3 ZnO NP effect on bacterial cell morphology

The cellular morphology of both bacterial strains was determined before and after treatment by SEM. After treatment with different concentrations of ZnO NPs, bacterial cells were fixed with primary fixative reagents (glutaraldehyde 2.5%, paraformaldehyde 0.1 ml l^−1^ in sodium cacodylate buffer) and incubated for 1 h at 4 °C. After incubation, the bacterial samples were centrifuged for 5 min at 1000 rpm and washed thrice with ultrapure water (Milli Q). Furthermore, the samples were dehydrated with increasing concentrations of ethanol (10%, 20%, 30%, 40%, 50%, 75%, and 100%) in water. These dehydrated bacterial cell samples were dried in a vacuum oven below 50 °C. The dried materials were mounted on SEM stubs with a thin layer of carbon tape, followed by sputter-coating (Turbo Sputter Coater (K575X) Emitech, Kent, UK). Then, the stub-mounted samples were observed at low voltage by SEM (JEOL, Ltd., Tokyo, Japan).

### 2.8 Statistical analysis

In this study, each result is expressed as the mean ± standard error of triplicate independent experiments. Differences with *p* < 0.05 were considered statistically significant. Statistical analysis was performed by one-way ANOVA with Dunnett's post hoc test to compare values between the control and treated groups.

## 3. Results and discussion

### 3.1 Quantification of flavonoids and phenols and preparation of ZnO NPs

Plant leaves are a rich source of flavonoids and phenolic components that have the potential to trigger the reduction of Zn^2+^ and control the size of synthesized ZnO NPs. Here, we prepared an aqueous extract of *P. odorifer* leaves and quantified its flavonoids and phenolic components. It was observed that 0.105% of phenols (w/w) and 0.035% of flavonoids (w/w) were present in POLE. Free hydroxyl and carboxylic groups of the flavonoids or phenols present in the plant extract bind to the surface of Zn^2+^ and trigger the formation of ZnO NPs, while the C=O, C=O–C and C=C groups of heterocyclic compounds may act as a stabilizer.^50,51^ The concentration of the plant extract plays an essential role in the synthesis of stable ZnO NPs. In this study, we optimized the ratio of plant extract to zinc acetate at 50 ml of zinc acetate solution (20 mM) to 20 ml of the leaf extract (50 mg ml^−1^) for the preparation of ZnO NPs. The resulting solution turned a dark brown. ZnO NPs were also synthesized using 5–15 ml of the leaf extract. However, the resulting yield of ZnO NPs obtained was much lower. This might be due to insufficient quality of flavonoids and phenolic components present in 5–15 ml of the leaf extract to completely reduce the zinc acetate solution (20 mM) into ZnO NPs (data not shown). Furthermore, the quality of flavonoids and phenolic components present in 20 ml or more of the plant extract was sufficient to reduce all Zn^2+^ ions in the reaction mixture using a hot plate with continuous stirring at 80 °C. In this study, we cost-effectively synthesised ZnO NPs *via* a green synthesised route with reduce chemical toxicity.

### 3.2 Characterization of ZnO NPs

**Fig. 1:**
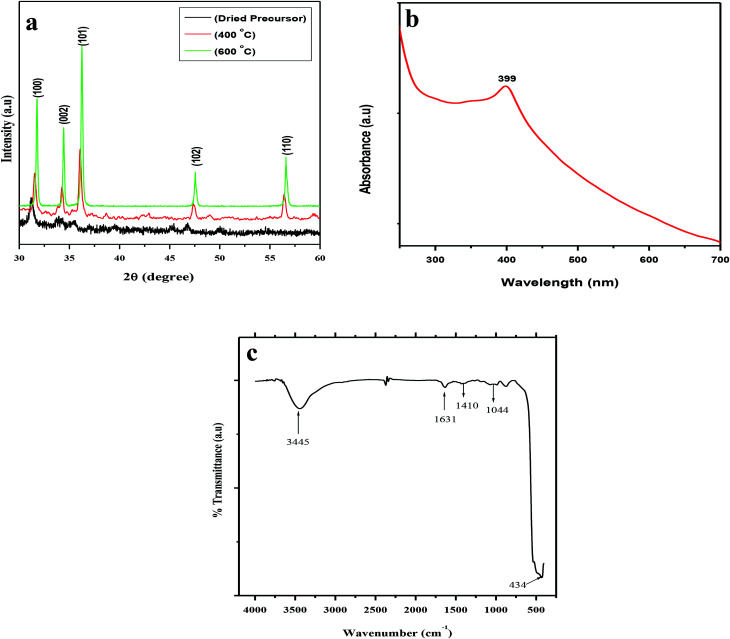
(a) Typical X-ray diffraction (XRD) patterns of biogenic precursor and biogenic ZnO NPs after calcination at 400 °C and 600 °C. (b) UV-vis spectrum of biogenic ZnO NPs after calcination at 600 °C. (c) FTIR spectrum of biogenic ZnO NPs after calcination at 600 °C.

The XRD patterns of the dried precursor (template hybrids) at room temperature and the sintered ZnO product at various temperatures were determined and are shown in Fig. 1a. The XRD patterns were very well matched with JCPDS, 36-1451, indicating that all the diffraction peaks of the sintered samples showed the monophasic zincite structure of ZnO NPs. The XRD patterns demonstrate 2θ values at 31.74°, 34.38°, 36.22°, 47.50°, and 56.54° which corresponded to the crystal planes, *i.e.*, (100), (002), (101), (102), and (110), thus confirming the presence of ZnO. Most of the peaks belong to the single phase of ZnO and impurity peaks were not observed, which indicates the high purity of the ZnO NPs. This indicates the crystalline nature of synthesized nanoparticle which was in agreement with the earlier reports using *Plectranthus Amboinicus* leaf extract synthesis of ZnO NPs.^52^ Our results are comparable to Ishwarya *et al.*^53^ reported the synthesis of ZnO nanoparticles using Ulva lactuca seaweed extract and, Narendhran *et al.*,^54^ who fabricated zinc nanoparticles using the *Lantana aculeate* leaf extract, while Vanathi *et al.*,^55^ synthesized the nanoparticles using *Eichorrnia crassipes* leaf extract.

The dried precursor was mainly amorphous because of the biological functional groups as organic components. The existence of weak ZnO peaks indicates that little crystalline ZnO is formed in the solution at room temperature. After calcination of the precursor at 400 °C, there is evident crystallization. The calcination process occurs at 500 °C, and as the intensities of the main peaks are enhanced, new patterns of diffraction peaks appear. The diffraction peaks become sharper with an increase in the calcination temperature to 600 °C, suggesting that the integrity of the crystalline structure increased. No characteristic peaks of any impurities were detected, which demonstrates that the product has a high phase purity.

UV-vis spectroscopy is a widely used technique to characterize the optical properties of synthesized NPs. Fig. 1b represents the UV-vis absorption spectra of the biosynthesized spherical ZnO NPs at room temperature. The characteristic absorption spectrum of ZnO shows a well-defined exciton band at ∼399 nm (calculated band gap of ∼3.10 eV), which is very close to the bulk exciton absorption of ZnO (373 nm).^56,57^ Due to the presence of a broad peak in the UV-vis spectra, the grown ZnO NPs showed excellent optical properties. In this study, the appearance of a single peak at approximately 399 nm indicated the formation of spherical ZnO NPs ∼90 nm in size.

The FTIR spectra further supported the formation of ZnO NPs using aqueous POLE and calcination at 600 °C. The FTIR spectra of the spherical ZnO NPs biosynthesized with the help of POLE are presented in Fig. 1c. The spectra show a very broad and intense band at 3445 cm^−1^ associated with the stretching vibration of the –OH (hydroxyl) and –NH (amine) groups of POLE. The characteristic peak at 1631 cm^−1^ can be attributed to the C=O (carbonyl) groups. The absorption band at 1410 cm^−1^ and 1044 cm^−1^ could be attributed C–C and C–N stretching respectively. The strong absorption band at 434 cm^−1^ is characteristic of ZnO NPs.^52,58^*P. odorifer* extract was also considered as the capping ligands, which give stability to the nanoparticles.^42^

**Fig. 2:**
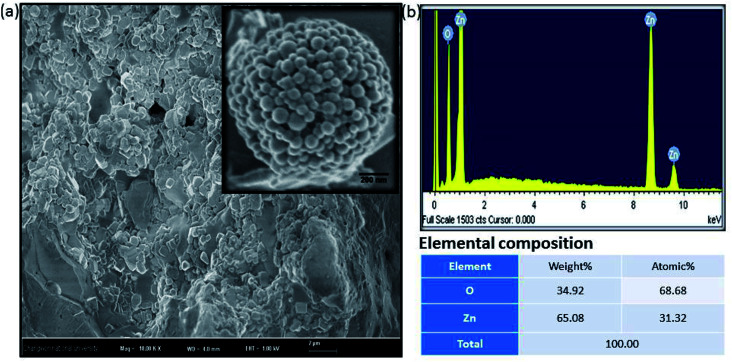
Amorphous biogenic ZnO materials (a) FESEM image with high magnification (b) their corresponding EDX spectrum with elemental composition.

Fig. 2a shows a FESEM image of the calcined ZnO NPs. The diameter of the spherical ZnO nanocrystals was ∼90 nm, as determined by FESEM. The elemental composition of the ZnO nanocrystals was investigated using EDX. The EDX plot as shown in Fig. 2b depicts the peaks of Zn and O for the ZnO calcined at 600 °C, which indicates that the ZnO structures are a combination of only Zn and O, as shown in Fig. 2b. No evidence of other impurities was found, which also confirms the high purity of the ZnO nanocrystals.

### 3.3 Anticancer activity of biosynthesized ZnO NPs

#### 3.3.1 MTT assay and cellular morphology

**Fig. 3:**
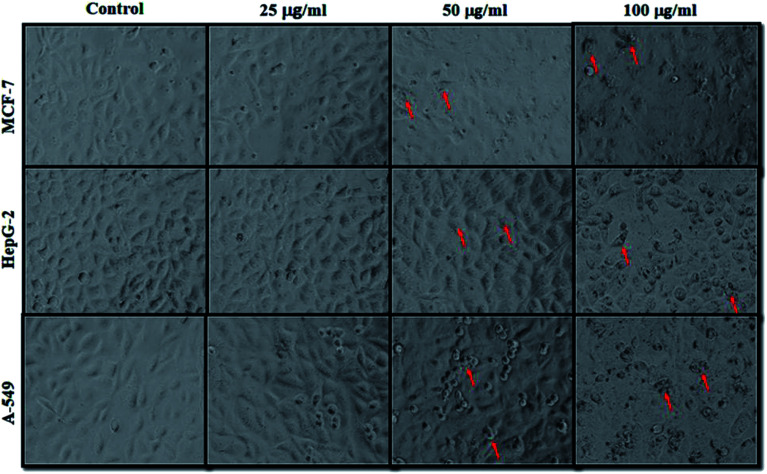
Change in morphological structure of MCF-7, HepG-2 and A-549 cells following the exposure of variable dose of ZnO nanocrystals for 24 h. Images were captured under the phase contrast inverted microscope at 20× magnification. *p < 0.05, **p < 0.001 *versus* control.

MCF-7, HepG2, and A-549 cells were exposed to the biosynthesized ZnO NPs for a period of 24 h, and the morphological changes were observed (Fig. 3). The cancer cells were treated with 1–100 μg ml^−1^ of ZnO NPs, and the cell proliferation was examined using an inverted phase contrast microscope. Furthermore, cell viability was determined by MTT assay.^59^ In the mitochondria of living cells, yellow MTT solution is reduced to purple formazan salt. Consequently, DMSO (solubilization buffer solution) is added to dissolve the insoluble purple formazan product into a coloured solution. The cell viability in coloured solution was at 550–570 nm determined using a spectrophotometer.

The maximum absorption depends on the solvent employed, and the percentage (%) viability was calculated according to the following equations
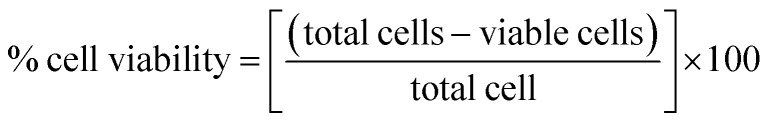
or
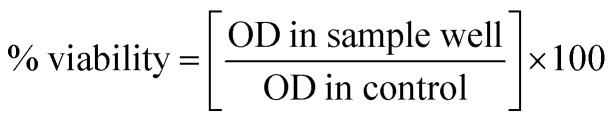


**Fig. 4:**
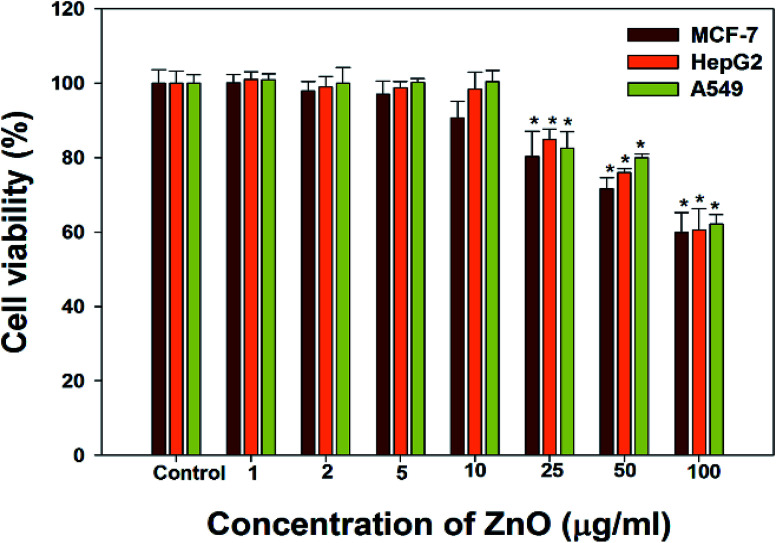
Cytotoxicity in MCF-7 cells; HepG2 cells; and A549 cell detection by MTT assay. Cells were exposed to different dose (1–100 μg ml^−1^) ZnO for 24 h. Each data values are mean ± SE of three independent experiments. *p < 0.05, **p < 0.001 *versus* control.

The cancer cells (MCF-7, HepG2, and A549) were treated with different doses of ZnO NPs (1–100 μg ml^−1^) for 24 h, and the results are presented in Fig. 4. We found that the viability of cells decreased with increasing concentrations of ZnO NPs. Cell viability was observed in the range of 80–100% in after treatment with ZnO NPs ranging from 1–25 μg ml^−1^ in concentration. Conversely, at two higher concentrations, *i.e.*, 50 and 100 μg ml^−1^, the viability of all studied cancer cells was reduced to only 70% and 60%, respectively (Fig. 4). The observed reductions in cell viability at higher ZnO NP doses were statistically significant (*p* < 0.05).

#### 3.3.2 NRU assay

**Fig. 5:**
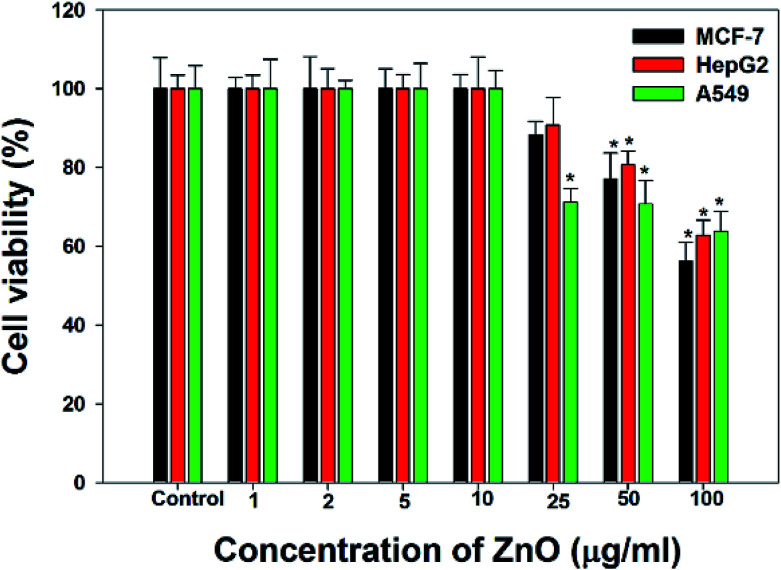
Cytotoxicity in MCF-7 cells; HepG2 cells; and A549 cells during neutral red uptake (NRU) assay. All the cells were exposed to different dose (1–100 μg ml^−1^) of ZnO for 24 h. Values are mean ± SE of three independent experiments. *p < 0.05, **p < 0.001 *versus* control.

To support the anticancer study, NRU assay was performed on MCF-7, HepG2 and A549 cells using different doses of ZnO NPs (Fig. 5). The same pattern of cell viability was observed in the NRU assay as in the MTT assay. We observed a statistically significant (*p* < 0.05) decrease in the viability of MCF-7, HepG2, and A549 cancer cells after treatment with 50 and 100 μg ml^−1^ ZnO. At ZnO NP concentrations, less than 50 μg ml^−1^, no significant effect of was observed on the viability of the studied cancer cells.

#### 3.3.3 Effect of biosynthesized ZnO NPs in cancer cell apoptosis determined by flow cytometry

**Fig. 6:**
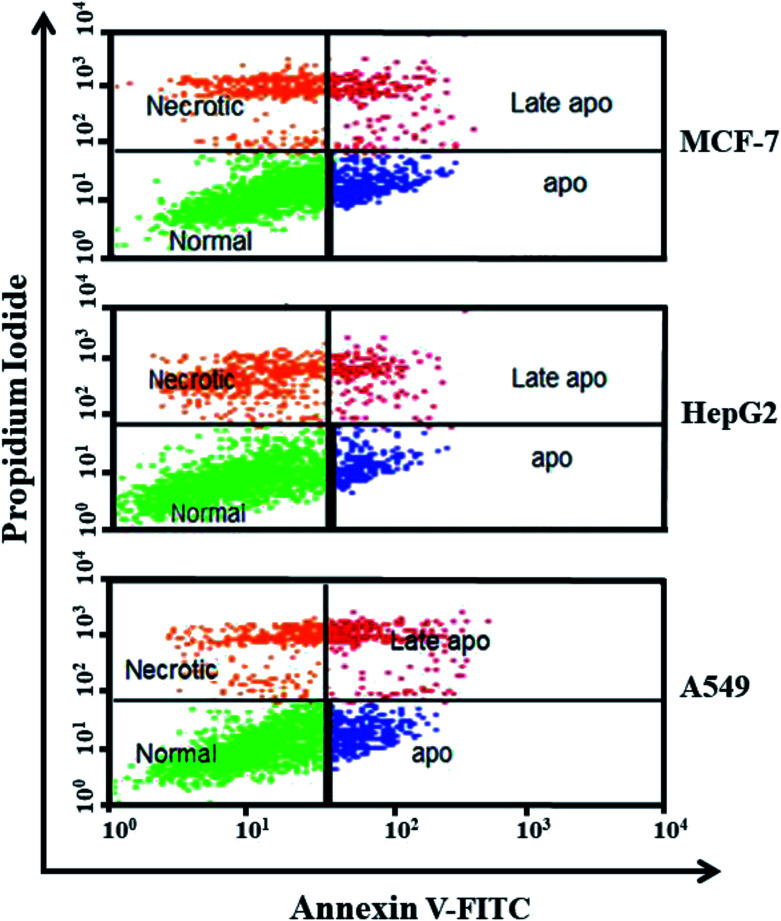
The apoptotic effect of biosynthesized ZnO NPs at 100 μg ml^−1^ concentration after 48 h incubation, using Annexin V-FITC/PI staining of tested MCF-7, HepG2, and A549 cancer cell line. Here dots represent cells as follows: lower right quadrant, early apoptotic cells (FITC^+^/PI^−^); lower left quadrant, normal cells (FITC^−^/PI^−^); upper right quadrant, late apoptotic cells (FITC^+^/PI^+^); upper left quadrant, necrotic cells (FITC^−^/PI^+^).

Apoptosis and necrosis are the main mechanisms of cell death. In apoptosis, cells are induced to commit programmed death because of a response to internal or external stimuli, while, in necrosis, the cells are damaged by external injury. Many NPs have been reported to stimulate apoptosis in pre-malignant and malignant cells and hence act as anticancer agents.^60,61^ In the present study, flow cytometry was used to analyse the apoptosis and necrosis of MCF-7, HepG2 and A549 cancer cells after treatment with 100 μg ml^−1^ ZnO NPs for 48 h and staining with Annexin V and PI. Fig. 6 indicates a decrease in the viability of cancer cells after treatment with ZnO NPs, with MCF-7, HepG2, and A549 cells showing 60%, 62%, and 64% viability, respectively. Among the MCF-7 cells, 15.5%, 16.23%, and 8.27% were, apoptotic, necrotic and late apoptotic cells respectively, which resulted in approximately 40% total cell death. HepG2 cells were also exposed to the biosynthesized ZnO NPs for the same time and exhibited approximately 38% cell death, which was a cumulative result of 10.23% apoptosis and 22.19% necrosis followed by 6.58% late apoptosis. The lowest amount of cell death was found in A549 cancer cells, which was 36%, the sum of 13.12% apoptotic cells, 14.31% necrotic cells and 8.67% late apoptosis. We found that ZnO NPs were the most effective against MCF-7 cells.

Due to the unique biological properties of nanoparticles, it gets a tremendous approach for the treatment of diseases. The anticancer activity of ZnO NPs against human carcinoma cells has already been reported.^53^ In the present study, at higher concentrations of ZnO NPs, strong anticancer activity was observed. There were no significant morphological changes after treatment with the lower concentrations (1–10 μg ml^−1^) of ZnO NPs, but the growth of cancer cells decreased with increasing ZnO NP doses up to 100 μg ml^−1^. The cells were damaged at the two highest doses (50 μg ml^−1^ and 100 μg ml^−1^) of NPs (Fig. 3). The typical morphology and adhesion capacity of the treated cells compared to the controls were both reduced at high concentrations of ZnO NPs. The inhibition of MCF-7, HepG2, and A-549 cancer cell growth was found at higher concentrations, *i.e.*, 50 and 100 μg ml^−1^ of biosynthesized ZnO NPs. This was in close proximity to the findings of Ishwarya *et al.*^53^ reported 50% reduction of MCF-7 breast cancer cells were exhibited at 50 μg ml^−1^ of ZnO NPs while Selvakumari *et al.*^62^ who reported that 50% reduction of human A549 lung cancer cells and MCF-7 breast cancer cells were exhibited at 31.2 μg ml^−1^ of ZnO NPs. At a very low concentration, ZnO NPs exhibit activity against liver cancer HepG2 cells in a dose-dependent manner. At 25 μg ml^−1^, the viability of HepG2 cells was less than 10%.^63^ In the present study, a significant reduction in the cell viability in the cancer cells were observed through apoptosis, necrosis and late apoptosis which resulted the decrease in the viability of cancer cells after treatment with ZnO NPs, with MCF-7, HepG2, and A549 cells showing 60%, 62%, and 64% viability at 100 μg ml^−1^, respectively. These findings are in agreement with Sanaeimehr *et al.*^64^ who reported the 50% cell viability at 175 μg ml^−1^ in HepG2 cancer cell. Boroumand Moghaddam *et al.*^65^ found IC_50_ value was 121 μg ml^−1^ for MCF-7 cells. This suggests that the biosynthesized ZnO NPs have the potential to treat the breast, lung and liver cancer without any harmful effect.

### 3.4 Antimicrobial activity

#### 3.4.1 Bacterial growth inhibition in the presence of ZnO NPs

**Fig. 7:**
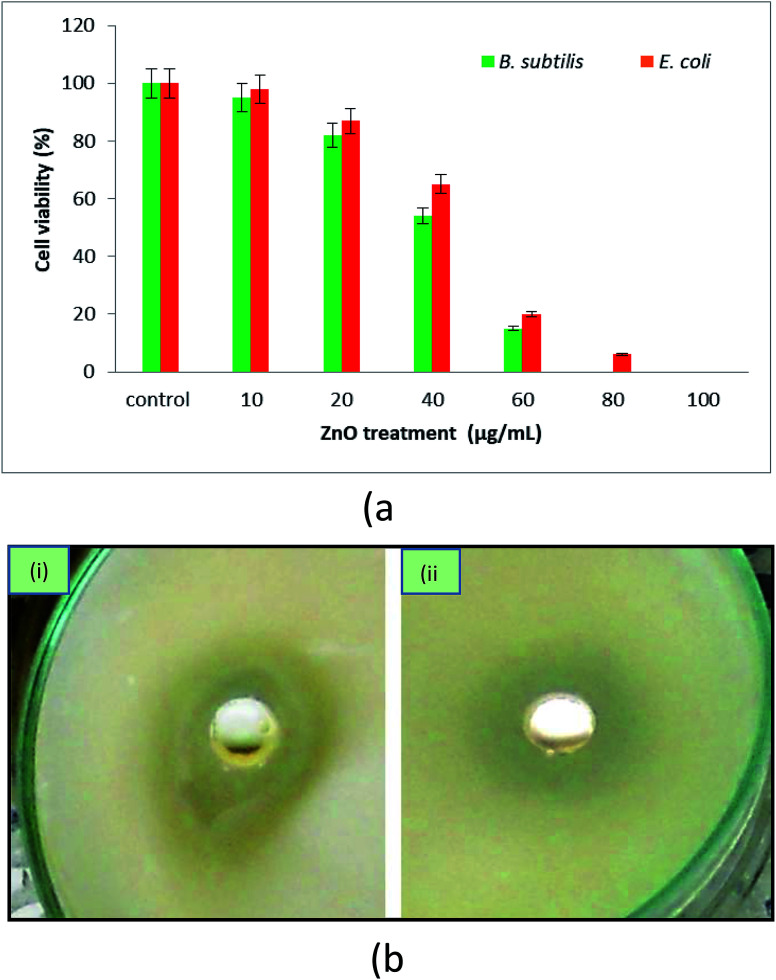
(a) Effect of ZnO NPs on the growth of Gram-positive (*B. subtilis*) and Gram-negative (*E. coli*) bacteria. (b) Zone inhibition image of (i) *B. subtilis* and (ii) *E. coli* in the presence of biogenic ZnO loaded in the wells of medium plate.

The effect of biosynthesized ZnO NPs on the growth of Gram-positive and Gram-negative bacteria was studied at different concentrations by the broth-dilution method as well as by plating on agar plates. We found that increasing concentrations of ZnO NPs had a significant inhibitory effect on the growth of both bacterial strains (Fig. 7a). However, the bacteriostatic effect was more prominent on the Gram-positive bacterial strain (*B. subtilis*) than the Gram-negative bacteria strain (*E. coli*). We observed that 85% of *B. subtilis* and 80% of *E. coli* growth was inhibited by treatment with 60 μg ml^−1^ ZnO NPs. The maximum inhibitory concentration was observed to be 80 μg ml; at this concentration, the growth of Gram-positive bacteria completely vanished, while more than 95% of the growth of Gram-negative bacteria was also inhibited. Minimum inhibitory concentration (MIC) and minimum bactericidal concentration (MBC) of the green synthesized ZnO NPs ranged from 40–60 g ml^−1^ and 80–100 g ml^−1^, respectively, against the Gram-positive and Gram-negative bacterial strains. It is significant to note that the growth of both *B. subtilis* and *E. coli* was significantly inhibited by ZnO NPs at least at 40–80 μg ml^−1^, which is in agreement with the findings of previous studies on biologically synthesized ZnO NPs.^66^ Similarly, the bactericidal activity of the ZnO NPs against *Campylobacter jejuni*, *Salmonella enterica*, and *Escherichia coli* has been previously reported.^67–69^ The bactericidal effect of the ZnO NPs might be attributed to disruption of the bacterial cell membrane.^67^ Furthermore, it has been observed that the shape and size of NPs also play decisive roles in determining the bactericidal activity of NPs.^70^ Although many studies have reported the biogenic synthesis of ZnO NPs and their antimicrobial activity, this is the first study wherein ZnO NPs were synthesized *via* a green route involving POLE and their antimicrobial and anticancer activities were explored.

#### 3.4.2 Antibacterial activity of ZnO determined by zone inhibition assay

The biogenic ZnO NPs showed excellent antimicrobial activity against *B. subtilis* and *E. coli*. Zone inhibition tests were performed on solid nutrient agar media plates. Each plate contained a well loaded with the ZnO nanomaterial, which diffused into the surrounding media and prevented bacterial growth in a zone around the well. We observed excellent zone inhibition at 26 nm and 24 mm against *Bacillus subtilis* and *E. coli*, respectively, at a biogenic ZnO concentration of 50 μg per well (Fig. 7b). Similarly, in previous studies, inorganic/biogenic ZnO nanomaterial was also used as an antibacterial agent.^66,68^ During zone inhibition assay, nanomaterials stored in the wells of bacterial culture inoculated plate, here nanomaterials release and diffuse ions into the surrounding media where these ions interact with the inoculated bacteria and significantly prevent the growth of bacteria around the wells and develop a clear halo.

#### 3.4.3 Effect of ZnO NPs on bacterial cell morphology

**Fig. 8:**
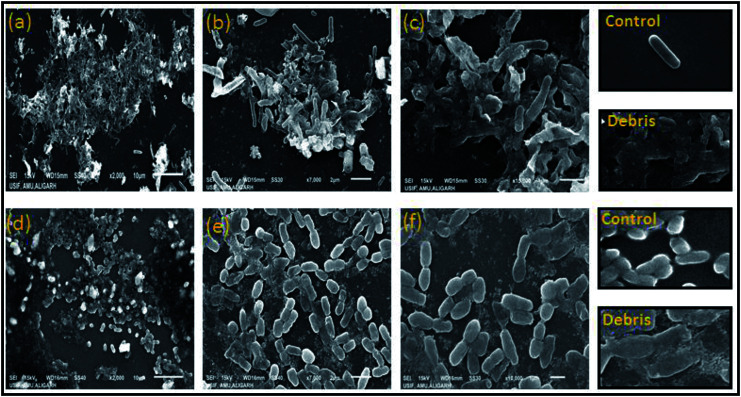
Scanning electron microscopy imaging at different resolution of partial damage and distorted cells of both bacterial cultures of *E. coli* at 2000× (a) 7000× (b) 15 000 (c) and *B. subtilis* at 2000× (d) 7000× (e) and 15 000× (f), treated with 75 μg ml^−1^ ZnO NPs in growing media and after 8 h incubation at 37 °C.

Previously, we observed that the growth of both Gram-positive and Gram-negative bacteria was inhibited by increasing concentrations of ZnO NPs. We further explored the effect of the ZnO NPs on the cellular morphology of *B. subtilis* and *E. coli* by SEM (Fig. 8). The SEM images of *B. subtilis* and *E. coli* exposed to ZnO NPs at a concentration of 50 μg ml^−1^ ZnO revealed partial damage and distortion of the bacterial cells (Fig. 8). Images were captured at different magnifications ranging from 2000 to 15 000× using a low voltage (15 kV). Fig. 8a–c show *E. coli* cells, and Fig. 8d–f show *B. subtilis* cells. It was hypothesized that bacterial cells become stressed in the presence of NPs possibly due to an interaction between the NPs and the bacterial membrane lipid molecules. This interaction may lead to the generation of free radicals, which can damage the membrane transport system and hence further affect bacterial metabolism and growth. In previous studies, researchers have observed that the leakage of protoplasmic inclusions from bacteria is proportional to the amount of interaction with ZnO NPs.^71^ Recently, researchers and drug developers are focusing on biogenic ZnO nanomaterials manufacturing because of its ease of synthesis and environmentally friendly and effective biomedical application.

## 4. Conclusion

In the present research, we synthesized ZnO NPs from POLE *via* a green route. The synthesized NPs were characterized by various techniques, including XRD, FESEM, EDX, FTIR and UV-vis spectroscopy. We were able to synthesize very pure, spherical ZnO NPs. The focus of this study was to synthesize NPs with anticancer and antimicrobial activities. Hence, the anticancer activity of the synthesized ZnO NPs was evaluated in MCF-7, HepG2, and A549 cells by phase-contrast microscopy and MTT and NRU assays. Furthermore, the anticancer profile of the ZnO NPs was studied using Annexin V-FITC/PI staining and flow cytometry. The flow cytometry results indicated that apoptosis, necrosis and late apoptosis were the main causes of cell death. These biosynthesized ZnO NPs were significantly effective against the studied cancer cell lines. Additionally, the antimicrobial activity of the ZnO NPs was confirmed in Gram-positive (*Bacillus subtilis*) and Gram-negative (*Escherichia coli*) bacteria. Overall, the results of this study establish that the biogenic synthesis of ZnO NPs from POLE leads to the formation of very pure, spherical NPs with anticancer and antimicrobial properties. These NPs have the potential for development into promising chemotherapeutic treatments for cancer and bacterial multidrug resistance but require further investigation.

## Conflicts of interest

There is no conflict of interest.

## Supplementary Material
